# Stereotactic body radiotherapy (SBRT) in oligometastatic ovarian cancer (OMOC): a systematic review and meta-analysis of clinical outcomes and toxicity profiles

**DOI:** 10.3389/fphar.2025.1620922

**Published:** 2025-10-27

**Authors:** Mauro Francesco Pio Maiorano, Brigida Anna Maiorano

**Affiliations:** ^1^ Department of Interdisciplinary Medicine (DIM), Unit of Obstetrics and Gynecology, University of Bari “Aldo Moro”, Polyclinic of Bari, Bari, Italy; ^2^ Unit of Oncologic Gynecology, IRCCS “Giovanni Paolo II” Oncologic Institute, Bari, Italy; ^3^ Department of Medical Oncology, IRCCS San Raffaele Hospital, Milan, Italy

**Keywords:** ovarian cancer, oligometastatic ovarian cancer, ovarian cancer radiotherapy, advanced ovarian cancer survival, stereotactic body radiotherapy ovarian cancer, sbrt ovarian cancer, systematic review omoc, meta analysis omoc

## Abstract

**Introduction:**

Oligometastatic ovarian cancer (OMOC) represents a distinct clinical state with a limited metastatic burden, potentially amenable to local ablative strategies. Stereotactic body radiotherapy (SBRT) has emerged as a promising treatment in this context, offering high-dose precision with minimal toxicity. However, evidence of its role in OMOC remains fragmented.

**Methods:**

We conducted a systematic review and meta-analysis of studies evaluating SBRT in patients with OMOC, focusing on clinical outcomes, including local control (LC), progression-free survival (PFS), overall survival (OS), and grade ≥3 toxicities. Eligible studies were identified through a comprehensive search across PubMed, Embase, Scopus, and Cochrane Library up to March 2025. Data synthesis involved pooled analysis using random-effects models.

**Results:**

Eight retrospective or prospective studies, encompassing 594 patients, were included. The majority of patients had received at least two prior lines of therapy. SBRT was delivered to ≤5 lesions, commonly during systemic treatment-free intervals or maintenance with PARP inhibitors. One-year LC ranged from 86.7% to 94.4%, and 2-year LC ranged from 60.9% to 88.9%. Median PFS ranged from 7.4 to 15.0 months, and median OS from 21.0 to 43.0 months. Grade ≥3 toxicities were rare (0%–6.1%), and no treatment-related deaths were reported.

**Discussion:**

SBRT demonstrates favorable LC and survival outcomes in selected OMOC patients while maintaining a low toxicity profile, despite current evidence being descriptive and thus to be interpreted with caution. SBRT use during systemic treatment breaks or as a tool to control oligoprogressive disease under maintenance therapy suggests a potential role in extending treatment-free intervals. These findings support SBRT as a valuable component of a multidisciplinary approach to OMOC and underscore the need for prospective, context-specific trials to validate these results.

**Systematic Review Registration:**

https://www.crd.york.ac.uk/PROSPERO/view/CRD420251161822, identifer CRD420251161822.

## 1 Introduction

Ovarian cancer (OC) remains one of the most lethal gynecological malignancies, accounting for a significant proportion of cancer-related deaths in women (Momenimovahed et al., 2019). Its high mortality is primarily attributed to late-stage diagnoses and the frequent development of recurrent or resistant disease following standard treatment (Arora et al., 2024). Although advancements in surgical techniques and systemic therapies, particularly the introduction of anti-angiogenic therapy with bevacizumab and the integration of platinum-based chemotherapy and Poly (ADP-ribose) polymerases (PARP) inhibitors (PARPis), have improved short-term outcomes, the long-term prognosis for many patients remains poor, particularly upon relapse (Tuninetti et al., 2024; Maiorano et al., 2022). In this setting, attention has increasingly turned to the potential role of local therapies in selected patients, especially those with limited metastatic burden. The oligometastatic disease (OMD) concept, introduced by Hellman and Weichselbaum in 1995, describes an intermediate state between localized and widely disseminated cancer (Hellman and Weichselbaum, 1995). It is biologically distinct and potentially amenable to curative-intent local therapies (Reyes and Pienta, 2015). While this paradigm has been increasingly embraced across several solid tumors, such as non-small cell lung cancer, colorectal cancer, and prostate cancer, it remains poorly defined and under-investigated in OC (Couñago et al., 2019; Carconi et al., 2023; Jadvar et al., 2022). One of the main challenges lies in the absence of a standardized or universally accepted definition of oligometastatic ovarian cancer (OMOC). Across the available literature, the maximum number of lesions considered “oligo” ranges from three to five, with inconsistent criteria regarding anatomical site, lesion size, prior treatments, and disease-free interval (Ottaiano et al., 2023). This heterogeneity hampers cross-study comparisons and highlights the need for more structured clinical frameworks. Despite these limitations, there is growing evidence suggesting that a subset of patients with OMOC, particularly those with platinum-sensitive disease or oligoprogressive lesions under systemic control, may derive meaningful benefit from focal therapies. Stereotactic body radiotherapy (SBRT) has emerged as an attractive option among the available modalities (Lazzari et al., 2018). SBRT allows for delivering high-dose, highly conformal radiation over a limited number of fractions, maximizing tumoricidal effects while minimizing toxicity to adjacent healthy tissue (Guninski et al., 2024). The technique is well-suited to small-volume disease and has already demonstrated compelling outcomes in other oligometastatic contexts (Kinj et al., 2022). Recently, the concept of oligoprogression has gained increasing attention managing the metastatic disease, including OC. Oligoprogression refers to a clinical scenario in which a limited number of metastatic lesions (commonly defined as ≤3–5) exhibit progression while the remaining disease remains stable under systemic treatment (Cerda et al., 2022). This may occur either in patients with an overall oligometastatic burden or in those with otherwise polymetastatic disease under control. In both cases, focal ablative strategies—particularly SBRT—may be leveraged to target the progressing lesions, potentially delaying the need to switch systemic therapy and extending treatment-free intervals (Willmann et al., 2024). The application of SBRT in oligoprogressive settings aligns well with its precision and efficacy in controlling small-volume disease, further expanding its potential clinical role. Although several retrospective series have reported encouraging local control and survival outcomes with SBRT in OMOC, the evidence remains fragmented, and the clinical role of SBRT has yet to be clearly defined (Kowalchuk et al., 2020; Sherwani et al., 2023). In this systematic review and meta-analysis, we aim to synthesize and critically appraise the available literature on SBRT in OMOC, focusing on key clinical outcomes such as local control (LC), progression-free survival (PFS), overall survival (OS), and treatment-related toxicity. By consolidating the current body of evidence, this study seeks to clarify the therapeutic potential of SBRT in oligometastatic ovarian cancer and identify gaps to guide future prospective investigations.

## 2 Materials and methods

We registered this Systematic Review on PROSPERO (ID: CRD420251161822).

### 2.1 Search strategy

A comprehensive literature search was performed to identify studies evaluating the role of SBRT in patients with OMOC. Two reviewers (MFPM and BAM) independently searched, and any discrepancies were resolved by consensus. The databases searched included PubMed, Scopus, Embase, and the Cochrane Library, covering publications up to March 2025. The search strategy combined Medical Subject Headings (MeSH) and free-text keywords, including: “ovarian cancer,” “ovarian neoplasms,” “oligometastatic,” “oligorecurrent,” “oligoprogressive,” “stereotactic body radiotherapy,” “SBRT,” “radiotherapy,” “local treatment,” “surgery,” “PARPis,” “chemotherapy,” and “ablative therapy.” Boolean operators (AND/OR) were used to refine the search. In addition, the reference lists of included studies and relevant reviews were manually screened to identify any additional eligible publications not retrieved in the initial search.

### 2.2 Eligibility criteria

Studies were selected based on predefined criteria using the PICOS framework (Amir-Behghadami and Janati, 2020; Table 1). Eligible studies included those evaluating SBRT, either alone, used after or in combination with PARPi, CHT or surgery, in patients diagnosed with OMOC, defined as having a limited number of metastatic lesions (typically ≤5), and reporting extractable oncologic and safety outcomes, regardless of the number of prior lines of systemic therapies. We included randomized controlled trials (RCTs) and prospective or retrospective cohort studies, while excluding case reports, case series, reviews and commentaries.

**TABLE 1:** PICOS framework.

PICOS component	Definition
Population (P)	Adult patients with histologically confirmed ovarian cancer in the oligometastatic setting
Intervention (I)	Stereotactic body radiotherapy (SBRT), alone, after or in combination with PARPis, chemotherapy and/or surgery
Comparison (C)	Single-arm, prospective, or retrospective studies without comparators were included
Outcomes (O)	Primary outcomes: Local control (LC), progression-free survival (PFS), overall survival (OS). Secondary outcome: Grade ≥3 treatment-related toxicity
Study Design (S)	Prospective or retrospective studies including ≥10 patients and reporting quantitative clinical outcomes. Reviews, case reports, conference abstracts, and preclinical studies were excluded

### 2.3 Study selection and data extraction

The study selection followed the PRISMA (Preferred Reporting Items for Systematic Reviews and Meta-Analyses) guidelines (Page et al., 2021). Two independent reviewers (MFPM, BAM) screened the titles and abstracts of retrieved articles. Full-text versions of potentially eligible studies were reviewed for final inclusion. Discrepancies in study selection were resolved through discussion or consultation with a third reviewer if needed. Data extraction was performed using a standardized template. Extracted variables included: author, publication year, study design, sample size, patient characteristics, number and location of metastases, type and dose of radiotherapy, follow-up duration, and oncologic outcomes. Outcomes of interest included LC, PFS, OS, and toxicity (graded according to Common Terminology Criteria for Adverse Events [CTCAE] criteria (Freites-Martinez et al., 2021)).

### 2.4 Data synthesis and statistical analysis

Descriptive statistics were used to summarize the characteristics of the included studies. Given the observational nature of the evidence and the heterogeneity across studies (platinum sensitivity, clinical setting, and concomitant systemic therapy), pooled estimates in this review are intended as descriptive summaries of event frequencies, not comparative effectiveness measures. Therefore, all pooled results should be interpreted cautiously and as hypothesis-generating. A meta-analysis was conducted on a subset of studies reporting comparable quantitative outcomes for LC, PFS, OS, and toxicity. Proportions were pooled using a random-effects model (DerSimonian and Laird method) to account for inter-study heterogeneity (DerSimonian and Laird, 1986; DerSimonian and Kacker, 2007). The degree of heterogeneity was assessed using Cochran’s Q test and the I^2^ statistic, with values > 50% indicating substantial heterogeneity (Cochran, 1950). Time-to-event outcomes (PFS and OS) were synthesized using pooled medians and survival rates at defined time points (e.g., 1 and 2 years), where available. Forest plots were generated to visualize study-specific and pooled estimates. Statistical analyses were performed using R software (meta package, version 4.2.2) and SPSS version 24 (IBM Corp, 2016). Subgroup meta-analyses were pre-specified but not feasible because most studies did not report stratified numerators/denominators or comparable time points.

### 2.5 Risk of bias

To assess the methodological quality of the included studies, we applied the Newcastle–Ottawa Scale (NOS), a validated tool for evaluating the risk of bias in non-randomized studies (Wells et al., 2011). The NOS assesses studies across three domains: Selection (maximum 4 points), Comparability (maximum 2 points), and Outcome (maximum 3 points), for a total score out of 9. Two independent reviewers performed the assessment, and discrepancies were resolved by discussion and consensus. Based on the total score, studies were categorized as low risk of bias (7–9 points), moderate risk (5–6 points), or high risk (≤4 points).

## 3 Results

**FIGURE 1:**
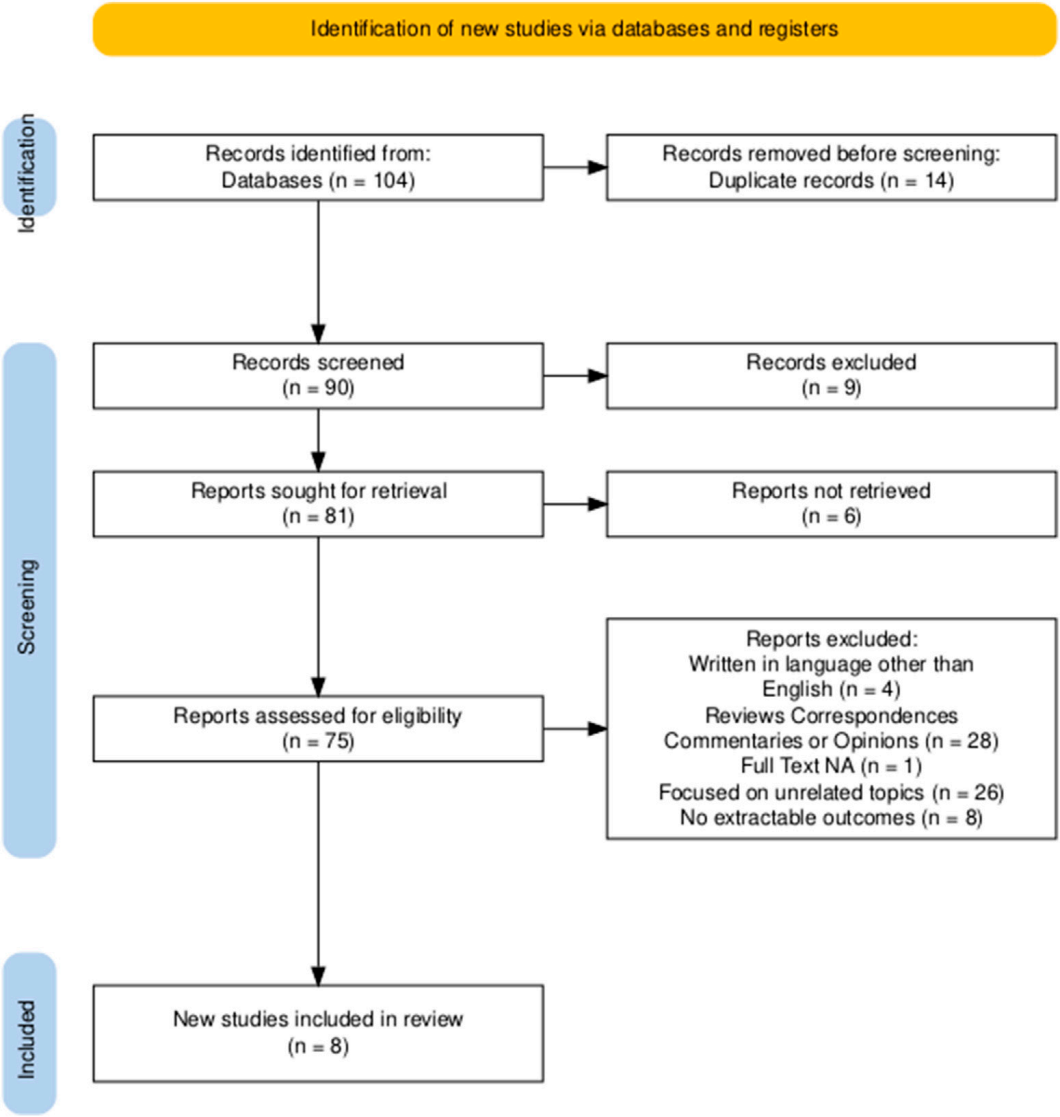
PRISMA flowchart for study selection.

A total of 104 records were identified through a systematic search of PubMed, Embase, Scopus, and the Cochrane Library. After removing 14 duplicates, 90 records were retained for title and abstract screening. Of these, 75 studies were selected for full-text evaluation. Following application of the predefined inclusion and exclusion criteria, 67 studies were excluded for the following reasons: 4 were written in languages other than English; 28 were reviews, correspondences, commentaries, or expert opinions; 1 study did not have full-text availability; 26 focused on unrelated topics, such as preclinical or molecular analyses without clinical endpoints; 8 did not report extractable outcomes on local radiotherapy for oligometastatic ovarian cancer. After the selection process, 8 studies met all inclusion criteria and were included in the final systematic review (Macchia et al., 2020; Shen et al., 2022; Onal et al., 2020; Iftode et al., 2018; Lazzari et al., 2018; Macchia et al., 2025a; Palluzzi et al., 2022; Macchia et al., 2025b). Figure 1 represents the PRISMA flowchart for study selection.

### 3.1 Characteristics of the included studies and overall main findings

This systematic review includes eight studies, all published between 2020 and 2025, focusing on the role of SBRT as a local ablative treatment in patients with oligometastatic or oligorecurrent OC. All studies were retrospective in design, except for one prospective observational cohort (Palluzzi et al., 2022). To contextualize the clinical utility of SBRT in OMOC, we systematically examined its therapeutic outcomes across diverse patient populations and treatment settings. Across the eight studies, a total of 594 patients were included. The number of patients per study ranged from 20 to 261. SBRT was employed in all cases, either as a standalone modality or in combination with systemic therapy such as chemotherapy or PARPis. No included study used surgery in the treatment of OMOC. The definition of oligometastatic disease varied slightly across studies, although a consistent threshold of ≤5 metastatic lesions was used in six studies. Two studies applied stricter criteria (≤3 lesions), particularly when SBRT was integrated during maintenance therapy or used to manage oligoprogressive disease. Some studies required all lesions to be technically suitable for SBRT (Shen et al., 2022; Macchia et al., 2025a), while others incorporated clinical performance status (e.g., ECOG 0–1 (Iftode et al., 2018; Macchia et al., 2020)). Some studies incorporated imaging-based metabolic criteria, such as FDG-PET positivity or volumetric assessment, to refine eligibility. For example, in Macchia et al., 2025a, both CT and FDG-PET were used to confirm lesion measurability and assess treatment response according to RECIST 1.1 criteria (Macchia et al., 2025b). None of the studies combined SBRT with surgical resection of metastases. Across the included studies, the median PFS (mPFS) ranged from 7.4 to 15.0 months, and the median OS (mOS), when reported, ranged from 21.0 to 43.0 months. Local control (LC) at 1 year ranged from 86.7% to 94.4%, and 2-year LC was reported between 60.9% and 88.9%. Grade ≥3 toxicity was rare, ranging from 0% to 6.1%, with no treatment-related deaths reported. These outcomes were seen in populations with both platinum-sensitive and platinum-resistant disease and patients treated with SBRT either as part of a treatment-free interval, during PARPi maintenance, or in chemo-free settings.

### 3.2 Patient population, previous therapies, and intervention

The included population consisted predominantly of patients with recurrent OC, either platinum-sensitive or platinum-resistant. Most patients had received ≥2 prior lines of systemic therapy before undergoing SBRT. For instance, in the largest study by Macchia et al., 2020, all patients had ≥2 prior lines of treatment, with many having had ≥3 lines (Macchia et al., 2020; Shen et al., 2022, enrolled patients with 2–4 prior lines, while Iftode et al., 2018, had a median of 2 prior systemic therapies (Iftode et al., 2018; Palluzzi et al., 2022, included patients receiving maintenance therapy with PARPis who developed radiologically confirmed oligoprogressive disease (Palluzzi et al., 2022). In this study, oligoprogression was defined as isolated progression (a single lesion), discrete progression (up to five lesions in different locations), or progression involving sanctuary sites such as brain or bone, while the remainder of the disease remained controlled. In Macchia et al., 2025b, oligoprogressive disease was defined as ≤5 progressing metastatic lesions during PARPi maintenance, while the remaining disease burden remained stable or responding (Macchia et al., 2025b). SBRT was delivered with curative or disease-controlling intent and typically targeted all measurable lesions when feasible. Most studies used modern image-guided techniques and hypofractionated schedules (e.g., 24–30 Gy in 3–5 fractions), though dose and fractionation schedules were variably reported. In Onal et al., 2020, SBRT was delivered during chemotherapy-free intervals as a strategy to delay re-initiation of systemic therapy (Onal et al., 2020); Macchia et al., 2025a applied SBRT either alone or in combination with ongoing systemic treatments (Macchia et al., 2025a). In contrast, Palluzzi et al., 2022 and Macchia et al., 2025b, delivered SBRT during active PARPi therapy to control oligoprogression without interrupting maintenance treatment (Palluzzi et al., 2022; Macchia et al., 2025b). A comprehensive summary of patient demographics, disease characteristics, and prior treatments across all included studies is reported in Table 2.

**TABLE 2:** Main included patients’ characteristics.

Characteristic	n (%) or summary
Total number of patients	594 (across 8 studies)
Median age (range)	56–63 years (range across studies: 33–84)
ECOG performance status 0–1	513 (86.3%)
Platinum-sensitive (PS)	228 (38.1%)
Platinum-resistant (PR)	158 (26.6%)
Mixed PS/PR	209 (35.2%)
Patients progressing on PARPi	21 (3.5%)
Median prior lines of therapy	2 lines median (range 1–5); >85% ≥ 2 lines
Definition of OMOC	≤5 lesions (6 studies), ≤3 lesions (2 studies); all measurable by imaging
Definition of oligoprogression	- Isolated lesion - ≤5 lesions, distinct locations - Sanctuary site progression under systemic control
Lymph node metastases	429 (72.2%)
Liver metastases	158 (26.5%)
Peritoneal metastases	103 (17.3%)
Lung metastases	67 (11.2%)
Bone metastases	5 (0.8%)
Lesions treated with SBRT	1–3; ≤5 allowed
SBRT during systemic therapy	3 studies (38.5%), CHT or PARPis
SBRT alone	5 studies (61.5%)

### 3.3 SBRT efficacy and clinical outcomes

Across all studies, SBRT demonstrated excellent local control. SBRT achieved high lesion-level control across the included series, with 1-year LC 86.7%–94.4% and 2-year LC 60.9%–88.9%, while median PFS generally clustered around 10–15 months and OS around 21–43 months (see Table 3 for study-level data). The longest PFS (15.0 months) and OS (43.0 months) were reported by Shen et al., 2022; within that cohort, single-lesion status and achieving disease control/response (DCR/ORR) aligned with superior survival, which helps explain the top-line figures. In the largest series (Macchia et al., 2020), SBRT produced durable per-lesion LC (24-month 81.9%), and complete response and total dose >25 Gy predicted longer LC; CR was more likely in nodal lesions, smaller PTV (≤18 cm^3^), and with BEDα/β10 > 70 Gy. Onal et al., 2020 similarly found that post-SBRT complete response correlated with higher 2-year PFS and OS with no grade ≥3 toxicity reported. Iftode et al., 2018 also described high clinical response with no grade ≥3 events and noted that most failures were distant, underscoring strong lesion-level control by SBRT. Detailed per-study values (with CIs/time points) are provided in Table 3.

**TABLE 3:** Summary of included studies on oligometastatic ovarian cancer.

Study	Design	N Patients	Population	Definition of oligometastatic	SBRT context	LC (%)	mPFS (months)	mOS (months)	Toxicity (≥G3,%)
Macchia et al. (2020)–MITO RT1	Retrospective multicenter	261	PS/PR-ROC, ≥2 prior lines	≤5 lesions, controlled primary, ECOG ≤2	SBRT	1y: 92.5%, 2y: 86.2%	13.5	42.7	4.6%
Shen et al. (2022)	Retrospective single-center	40	PS-ROC, 2–4 prior lines	≤5 lesions, SBRT suitable	SBRT	1y: 94.4%, 2y: 88.9%	15.0	43.0	5.0%
Onal et al. (2020)	Retrospective	29	ROC	≤5 lesions, no systemic progression	SBRT + CHT	6m: 100%, 1y: 86.7%, 2y: 60.9%	13.0	31.0	3.4%
Iftode et al. (2018)	Retrospective	82	PS/PR-ROC; median 2 prior lines	≤5 lesions, ECOG 0–1	SBRT	1y LC: 94%	10.0	31.0	6.1%
Lazzari et al. (2018)	Retrospective	52	ROC; prior CHT	≤3 lesions, at least 6 months CHT-free	SBRT	NA	12.0	32.0	NA
Macchia, Campitelli et al. (2025a) - MITO-RT3/RAD	Retrospective	36	ROC; prior CHT	≤5 lesions, SBRT suitable	SBRT alone or + CHT	NA	10.0	21.0	NA
Palluzzi et al. (2022)	Observational	20	Progression on PARPis	≤3 lesions	SBRT + PARPi	NA	7.4	NA	0%
Macchia et al. (2025b) - EPIMETHEO	Retrospective	74	ROC undergoing PARPis (oligoprogression)	≤5 lesions, FDG-PET or MRI-defined	SBRT + PARPi	NA	10.0	NA	NA

CHT, chemotherapy; ECOG, Eastern Cooperative Oncology Group performance status; FDG-PET, fluorodeoxyglucose positron emission tomography; G3, grade 3; LC, local control; mOS, median overall survival; mPFS, median progression-free survival; MRI, magnetic resonance imaging; NA, not available; OS, overall survival; PARPi, poly (ADP-ribose) polymerase inhibitor; PFS, progression-free survival; PR-ROC, platinum-resistant recurrent ovarian cancer; PS-ROC, platinum-sensitive recurrent ovarian cancer; ROC, recurrent ovarian cancer; SBRT, stereotactic body radiation therapy.

### 3.4 Safety outcomes

SBRT was generally well tolerated across the included studies. A total of six studies reported treatment-related adverse events using CTCAE criteria, allowing for a consistent evaluation of safety outcomes. In Macchia et al., 2020, 12 out of 261 patients (4.6%) experienced grade ≥3 toxicity, with the most commonly reported events being fatigue and abdominal pain; notably, one patient developed radiation pneumonitis (Macchia et al., 2020; Shen et al., 2022, reported two grade ≥3 events (5.0%) among 40 patients, predominantly consisting of fatigue and gastrointestinal discomfort, though no treatment discontinuations or deaths were observed (Shen et al., 2022; Onal et al., 2020, observed one case of grade ≥3 gastrointestinal toxicity (3.4%) in their 29-patient cohort, while Iftode et al., 2018, recorded five grade ≥3 adverse events (6.1%) among 82 patients, including fatigue, abdominal pain, and one episode of grade 3 diarrhea (Onal et al., 2020; Iftode et al., 2018). In Palluzzi et al., 2022, no grade ≥3 adverse events were reported among 20 patients, and only one patient experienced grade 2 fatigue (Palluzzi et al., 2022). Importantly, none of the studies reporting adverse events documented any treatment-related deaths, underscoring the general safety of SBRT in this context. Table 3 summarizes the main findings from the included studies.

### 3.5 Risk of bias assessment

Among the eight studies included in this systematic review, two were assessed as having low risk of bias (Macchia et al., 2020; Iftode et al., 2018), five as having a moderate risk (Shen et al., 2022; Onal et al., 2020; Lazzari et al., 2018; Macchia et al., 2025a; Macchia, et al., 2025b), and one study (Palluzzi et al., 2022) was judged to have a high risk of bias due to limitations in patient selection, comparability, and outcome reporting. Most studies demonstrated adequate selection of patient cohorts and clearly defined interventions. However, comparability between groups was often limited due to the retrospective design and absence of control arms. Outcome assessment was generally robust, though the length of follow-up and detail of adverse event reporting varied. Overall, the quality of evidence was consistent with the observational nature of the available data (Table 4).

**TABLE 4:** Risk of bias assessment (Newcastle–Ottawa Scale). The green dot represents a low risk of bias, the yellow and red a moderate and high risk, respectively.

Study	Selection (max 4)	Comparability (max 2)	Outcome (max 3)	Total score (max 9)	Risk of bias
Macchia et al. (2020)	4	2	3	9	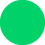
Shen et al. (2022)	3	1	2	6	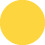
Onal et al. (2020)	3	1	2	6	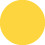
Iftode et al. (2018)	4	2	3	9	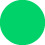
Lazzari et al. (2018)	3	1	2	6	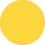
Macchia et al. (2025a)	3	1	2	6	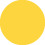
Macchia et al. (2025b)	3	1	2	6	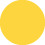
Palluzzi et al. (2022)	2	1	1	4	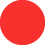

### 3.6 Meta-analysis of efficacy and safety outcomes

**FIGURE 2:**
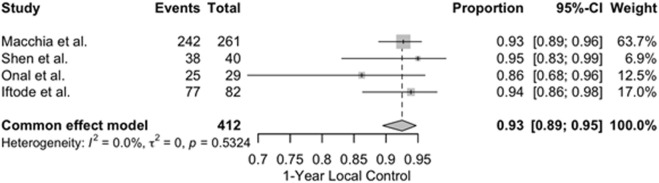
Forest plot of 1-year local control (LC) rates following stereotactic body radiotherapy (SBRT) in oligometastatic or oligorecurrent ovarian cancer across four studies (Macchia et al., 2020; Shen et al., 2022; Onal et al., 2020; Iftode et al., 2018). The pooled 1-year LC rate was 93% (95% CI, 89%–95%), with no significant heterogeneity observed among studies (I^2^ = 0%), supporting the use of a fixed-effect model. Individual study estimates ranged from 86% to 95%, highlighting the consistent efficacy of SBRT in achieving local tumor control across varied patient populations and treatment settings.

**FIGURE 3:**
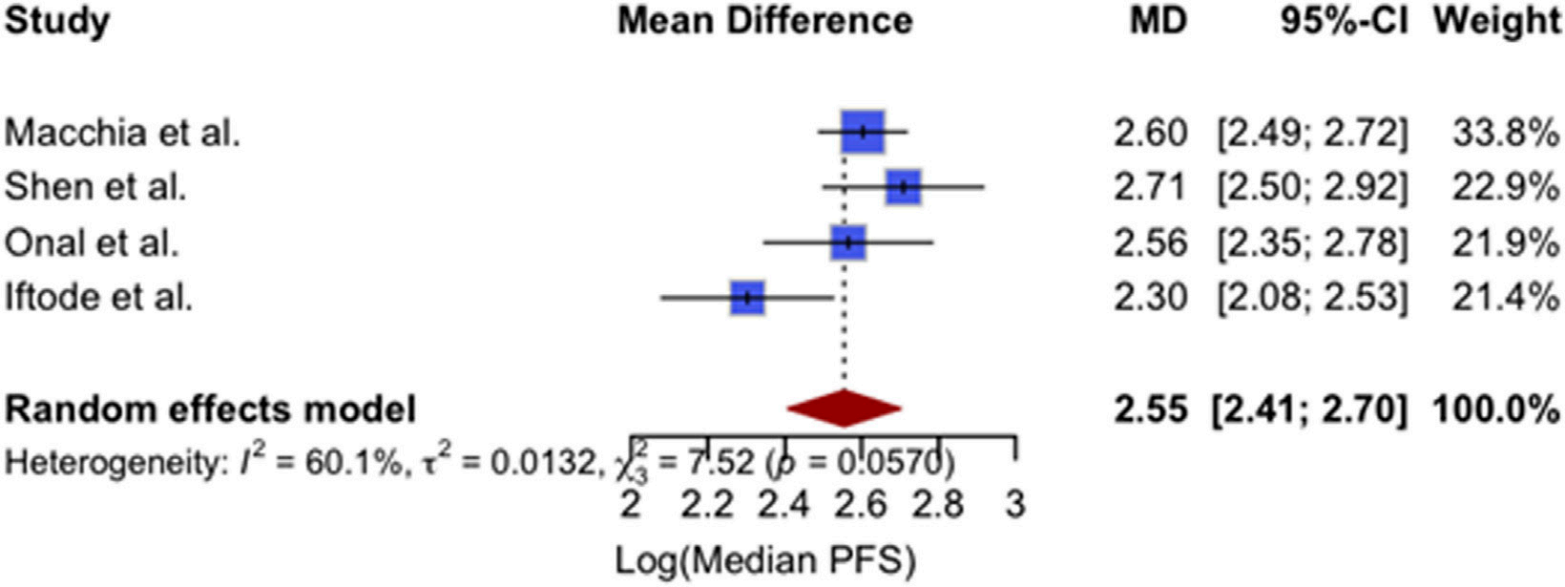
Forest plot of pooled log-transformed median progression-free survival (PFS) following SBRT in oligometastatic or oligorecurrent ovarian cancer. Data from four studies (Macchia et al., 2020; Shen et al., 2022; Onal et al., 2020; Iftode et al., 2018) were analyzed using a random-effects model. The pooled mean difference was 2.55 (95% CI, 2.41–2.70), with moderate heterogeneity observed (I^2^ = 60.1%).

**FIGURE 4:**
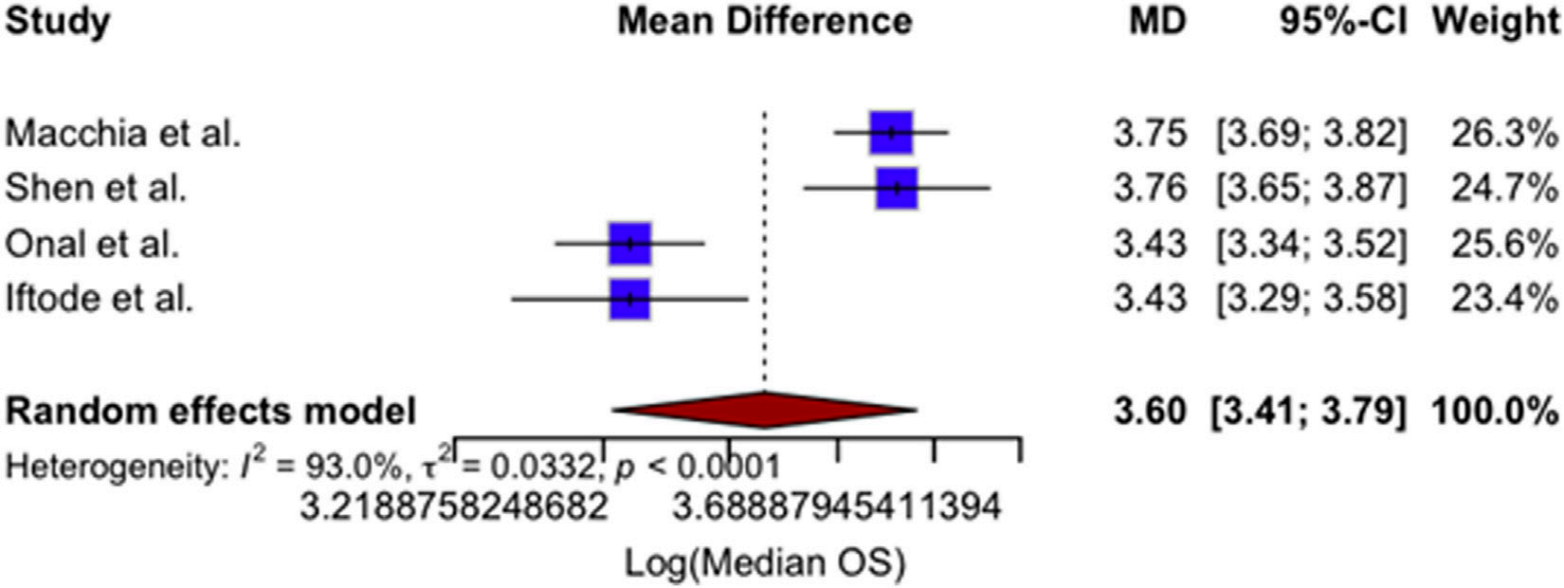
Forest plot of pooled log-transformed median overall survival (OS) after SBRT in oligometastatic or oligorecurrent ovarian cancer. Data from four studies (Macchia et al., 2020; Shen et al., 2022; Onal et al., 2020; Iftode et al., 2018) were combined in a random-effects model. The pooled mean difference was 3.60 (95% CI, 3.41–3.79), with substantial heterogeneity among studies (I^2^ = 93.0%, p < 0.0001).

**FIGURE 5:**
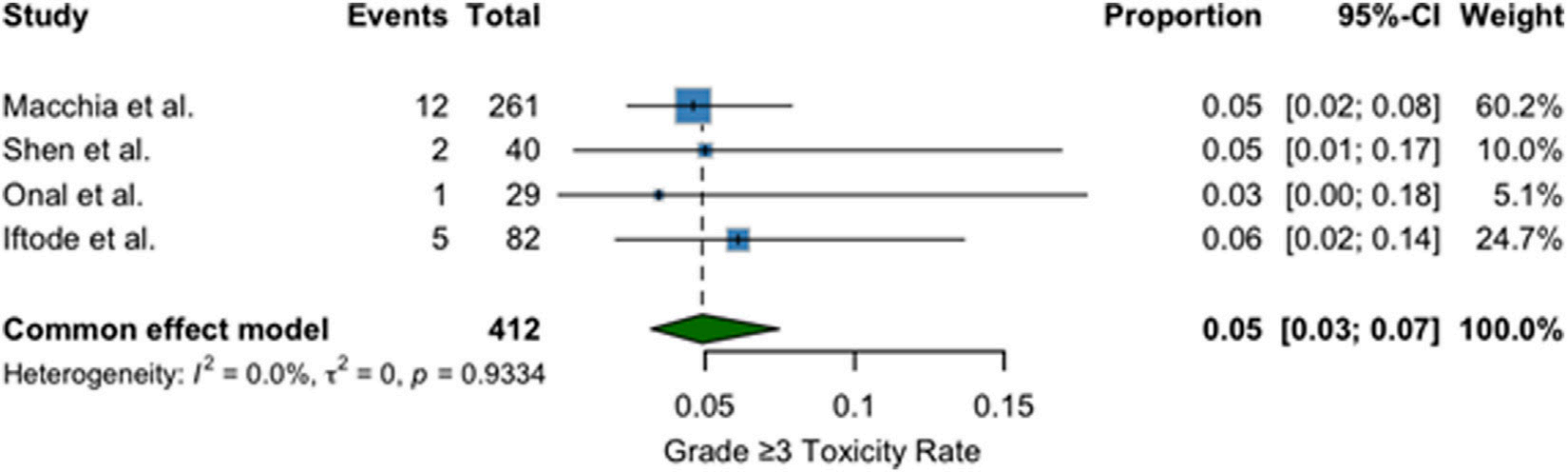
Forest plot of pooled proportion of grade ≥3 toxicity following SBRT in oligometastatic or oligorecurrent ovarian cancer. The pooled toxicity rate from 412 patients across four studies (Macchia et al., 2020; Shen et al., 2022; Onal et al., 2020; Iftode et al., 2018) was 5% (95% CI, 3%–7%), with no significant heterogeneity (I^2^ = 0%). A fixed-effect model was applied.

This section presents a descriptive juxtaposition of outcomes reported in different clinical contexts. It is not a comparative analysis, and no causal inferences should be drawn given the risk of confounding by indication and differences in systemic-therapy timing during SBRT. A quantitative synthesis of four studies reporting 1-year LC outcomes was performed, and the results are presented in Figure 2. The included studies comprised 412 patients treated with SBRT for oligometastatic or oligorecurrent OC (Macchia et al., 2020; Shen et al., 2022; Onal et al., 2020; Iftode et al., 2018). The pooled 1-year LC rate was 93% (95% confidence interval [CI]: 89%–95%), indicating excellent and consistent local tumor control following SBRT across diverse populations and clinical settings. No significant heterogeneity was observed between studies (I^2^ = 0%), supporting the appropriateness of a fixed-effect model. Individual study estimates ranged from 86% to 95%. A meta-analysis of four studies reporting mPFS following SBRT yielded a pooled mPFS of 12.9 months (95% CI: 11.1–14.9) (Macchia et al., 2020; Shen et al., 2022; Onal et al., 2020; Iftode et al., 2018). The analysis used a random-effects model due to moderate heterogeneity (I^2^ = 60.1%, p = 0.0570). Individual study estimates ranged from 10.0 to 15.0 months. Results are shown in Figure 3. A meta-analysis of four studies reporting mOS following SBRT demonstrated a pooled median OS of 36.7 months (95% CI: 30.2–44.3), as demonstrated in Figure 4. Due to the presence of significant heterogeneity (I^2^ = 93%, p < 0.0001), a random-effects model was used. Individual study estimates ranged from 31.0 to 43.0 months (Macchia et al., 2020; Shen et al., 2022; Onal et al., 2020; Iftode et al., 2018). A common-effect meta-analysis of four studies evaluating grade ≥3 toxicity following SBRT demonstrated a pooled incidence of 4.6% (95% CI: 3.1%–6.9%), with no significant heterogeneity (I^2^ = 0%, p = 0.74). Individual study estimates ranged between 3.4% and 6.1%, and no treatment-related deaths were observed (Macchia et al., 2020; Shen et al., 2022; Onal et al., 2020; Iftode et al., 2018). Results are summarized in Figure 5.

## 4 Discussion

This systematic review and meta-analysis provide a comprehensive synthesis of the current evidence regarding the efficacy and safety of local treatments, particularly SBRT, in patients with OMOC.

### 4.1 Oligometastatic ovarian cancer: a matter of definition

A fundamental challenge in interpreting the current literature on SBRT for OMOC lies in the lack of a standardized definition of oligometastatic disease. Originally conceptualized by Hellman and Weichselbaum as an intermediate state between localized and widely metastatic disease, the oligometastatic state has since been variably defined across clinical trials and retrospective studies, typically based on the number and location of metastatic lesions (Hellman and Weichselbaum, 1995). In this review, most included studies adopted a threshold of ≤5 metastatic lesions, while others applied stricter criteria (e.g., ≤3 lesions), particularly in the setting of oligoprogression or maintenance therapy (Lazzari et al., 2018; Palluzzi et al., 2022). Some studies also incorporated clinical performance status or imaging-based metabolic criteria to refine eligibility (Macchia et al., 2025b). This heterogeneity reflects the ongoing evolution of the oligometastatic concept, which is now increasingly recognized as a biologically distinct state rather than solely a numerical cutoff. Another critical consideration in the characterization of OMOC is the imaging modality employed to detect and quantify metastatic lesions. Although computed tomography (CT) remains the backbone of response assessment in ovarian cancer, several studies, including the EPIMETHEO study, utilized FDG-PET or PET/CT in conjunction with CT to refine eligibility and evaluate metabolic response (Macchia et al., 2025b). Given the superior sensitivity and specificity of PET in detecting small-volume or metabolically active disease, its use may uncover additional lesions not seen on CT, potentially reclassifying a patient from an oligometastatic to a polymetastatic status. This has substantial implications for treatment planning, patient selection, and comparability across studies, underscoring the need for standardized imaging protocols in future prospective trials (Qin et al., 2024). Emerging data suggest that factors such as tumor histology, genomic profiles, immune microenvironment, and the timing of metastatic spread (synchronous vs. metachronous) may all influence prognosis and treatment response (Belluomini et al., 2021). Consequently, efforts are underway to develop more refined classification systems, such as those proposed by ESTRO-ASTRO and EORTC, that incorporate clinical and biological parameters (Willmann et al., 2022). Until such frameworks are routinely implemented, caution is warranted when comparing outcomes across studies or extrapolating findings to broader patient populations.

### 4.2 SBRT and local control

Nonetheless, the consistently favorable results observed with SBRT in carefully selected OMOC patients reinforce the clinical utility of treating limited metastatic disease as a distinct and actionable therapeutic opportunity. Across the eight included studies, encompassing over 590 patients, SBRT emerged as a highly effective and well-tolerated local treatment strategy, achieving durable local control and encouraging survival outcomes even in heavily pretreated populations (Macchia et al., 2020; Shen et al., 2022; Onal et al., 2020; Iftode et al., 2018; Lazzari et al., 2018; Macchia et al., 2025a; Palluzzi et al., 2022; Macchia et al., 2025b). This finding is particularly notable in OMOC, a disease subset characterized by limited metastatic burden and a potentially indolent biology. Achieving durable LC in such patients is clinically meaningful, as it may delay systemic disease progression and prolong chemotherapy-free intervals. The LC rates observed in our analysis are consistent with those reported in large series of oligometastatic disease from other tumor types, including prostate, lung, and colorectal cancer, where SBRT has demonstrated 1-year LC rates ranging from 80% to 95% (Couñago et al., 2019; Carconi et al., 2023; Jadvar et al., 2022). Notably, SBRT outcomes in OMOC appear superior to those reported in other gynecologic malignancies, such as cervical cancer, where 2-year LC rates were as low as 62% in the MITO RT2 trial (Macchia et al., 2022a). These comparisons highlight the potential radiosensitivity of ovarian metastases and reinforce the role of SBRT as a modality capable of achieving robust LC. Additionally, response outcomes in several studies were associated with biologically effective dose (BED), with improved CR and LC rates observed in lesions treated with BED ≥70 Gy, underscoring the importance of dosimetric optimization in SBRT planning (Facondo et al., 2023).

### 4.3 SBRT and PFS: exploiting the abscopal effect

Regarding systemic disease control, the pooled mPFS of 12.9 months indicates that SBRT may offer clinically relevant delays in disease progression in carefully selected OMOC patients. While progression typically occurs outside of the irradiated fields, often due to the emergence of new metastases, the ability to postpone systemic relapse by approximately 1 year is particularly valuable in patients with limited treatment options, including those with platinum-resistant disease or those undergoing maintenance therapy. Notably, patients with lymph node-only oligoprogression or those receiving SBRT concurrently with PARP inhibitors appeared to derive particularly robust benefits, supporting the use of SBRT to extend systemic therapy duration without interruption (Macchia et al., 2025b). Although none of the included studies formally assessed immune-mediated responses, this phenomenon may relate to the so-called abscopal effect, wherein local radiotherapy exerts systemic anti-tumor activity beyond the irradiated sites (Reynders et al., 2015). The abscopal effect is hypothesized to be immune-mediated, involving the activation of cytotoxic T cells that target tumor cells at distant sites (Demaria and Formenti, 2020). While this effect has been most extensively documented in malignancies such as melanoma and non-small-cell lung cancer, emerging evidence suggests its potential relevance in OC (Nelson et al., 2023). For instance, a case report described a patient with oligometastatic platinum-resistant OC who achieved a partial response and sustained benefit for over 6 months following a combination of interstitial implantation radiotherapy, immunotherapy, and granulocyte-macrophage colony-stimulating factor (GM-CSF), suggesting a possible abscopal effect (Qin et al., 2024). These findings underscore the potential of combining SBRT with immunomodulatory agents to enhance systemic disease control in OMOC. These results align with PFS outcomes from randomized trials in other oligometastatic settings. For instance, the SABR-COMET trial reported an mPFS of 11.6 months in patients receiving SBRT for oligometastatic disease of various primary origins (Palma et al., 2020). Similarly, Gomez et al. (2019) demonstrated an mPFS of 14.2 months in oligometastatic non-small-cell lung cancer treated with local consolidative therapy, further validating the role of SBRT in extending disease control beyond the irradiated lesion (Gomez et al., 2019).

### 4.4 SBRT and OS: a beacon of hope for relapsed ovarian cancer?

The pooled mOS of 36.7 months reflects the potential of SBRT to meaningfully prolong survival in this rare patient population. This figure compares favorably to historical controls in relapsed OC, particularly in platinum-resistant settings, where OS rarely exceeds 12–18 months with systemic therapy alone (Hamontri and Tantitamit, 2023). Although the survival advantage of SBRT remains challenging to quantify in the absence of randomized data specific to OMOC, these results are on par with survival outcomes from SBRT-treated oligometastatic patients with other solid tumors. For example, SABR-COMET reported a median OS of 50 months with SBRT *versus* 28 months without (Palma et al., 2020), and similar trends have been observed in non-small-cell lung and renal cancers (David et al., 2024; Gomez et al., 2019). The favorable OS in our analysis likely reflects both patient selection (predominantly ECOG 0–1, median ≥2 prior treatment lines) and the ability of SBRT to provide durable local control without interrupting systemic maintenance or supportive care. Although formal patient-reported outcomes were not uniformly reported, the ability of SBRT to defer chemotherapy and prolong maintenance treatment likely translates into preserved quality of life, an important consideration in patients with cumulative treatment burdens. Importantly, the safety profile of SBRT in OMOC was reassuring.

### 4.5 Safety concerns

The pooled incidence of grade ≥3 adverse events was 4.6%, with no treatment-related deaths reported across more than 400 patients. Toxicities were generally mild and transient, with fatigue and gastrointestinal symptoms being the most frequently reported (Macchia et al., 2020; Shen et al., 2022; Onal et al., 2020; Iftode et al., 2018; Palluzzi et al., 2022). These findings align with the broader SBRT literature across various tumor types, where rates of severe toxicity typically range between 2% and 10%, depending on treatment site and prior therapies (Jia et al., 2023). The low incidence of high-grade toxicity in OMOC is particularly noteworthy given that most patients were heavily pretreated, and some received SBRT during ongoing systemic therapy (e.g., PARPis) without interruptions or exacerbations in adverse events (Macchia et al., 2025b). However, the potential for rare but serious complications, especially in cases of overlapping irradiation fields or abdominal targets, should not be underestimated and warrants careful planning and multidisciplinary decision-making.

### 4.6 Other OMOC local control modalities

Beyond SBRT, secondary cytoreductive surgery (SCS) remains a key option for carefully selected patients with platinum-sensitive first relapse. In the randomized DESKTOP III/ENGOT-ov20 trial, SCS followed by chemotherapy improved OS *versus* chemotherapy alone (median 53.7 vs. 46.0 months; HR 0.75; P = 0.02), with the greatest benefit observed when complete gross resection was achieved, supporting SCS as standard in centers with high complete-resection rates and robust selection pathways. (Harter et al., 2021). By contrast, GOG-0213 did not show an OS advantage for SCS in a setting where bevacizumab was frequently used, underscoring the importance of patient selection and likelihood of complete resection when considering surgery (Coleman et al., 2019). The Chinese SOC-1 trial further demonstrated a PFS benefit with SCS plus chemotherapy, reinforcing surgery’s role when complete resection appears feasible (Shi et al., 2021). In addition to surgery, other focal strategies can be considered in selected oligorecurrent scenarios, including salvage involved-field radiotherapy and image-guided ablation for liver or nodal disease, which have shown encouraging local-control and chemotherapy-free intervals in retrospective series (De Felice et al., 2017). Overall, these modalities complement SBRT within a multidisciplinary framework, with treatment choice driven by resectability, expected morbidity, lesion location, and institutional expertise.

### 4.7 Limitations and future directions

Despite the promising outcomes reported, several limitations must be acknowledged. This review pools predominantly retrospective studies with substantial clinical and methodological heterogeneity, including differences in platinum sensitivity, SBRT dose/fractionation, imaging/follow-up schedules, and whether systemic therapy was held or continued (e.g., during PARP-inhibitor maintenance). Because reporting was inconsistent, subgroup meta-analyses were not feasible without introducing selection bias. Accordingly, our pooled estimates are intended only to describe overall event frequencies and trends and should not be interpreted as comparative effectiveness across clinical contexts. Confounding by indication, center effects, and unmeasured prognostic factors likely influence PFS/OS and toxicity estimates, and publication/selection bias cannot be excluded. These constraints limit the precision and generalizability of our findings. The evidence should therefore be regarded as hypothesis-generating, highlighting a signal toward high local control and a potential clinical benefit of SBRT in OMOC settings that requires confirmation in prospective, context-specific studies. Furthermore, the definition of oligometastatic disease was not uniform across studies, and heterogeneity in SBRT dose, fractionation, and concurrent systemic therapy may have influenced outcomes. The absence of control arms and randomized comparisons also limits causal inference regarding the impact of SBRT on survival. Lastly, long-term follow-up data remain scarce, and the role of SBRT in combination with emerging systemic agents, such as immunotherapy or antibody-drug conjugates, has yet to be established. Nonetheless, this review offers essential insights into the potential role of SBRT as a safe and effective component of multimodal therapy in OMOC. The consistently high local control rates, favorable safety profile, and promising survival outcomes support the integration of SBRT in carefully selected patients, including those with platinum-resistant disease, oligoprogression under maintenance therapy, or contraindications to further systemic treatments. As prospective data from ongoing studies, such as MITO-RT3, become available (Macchia et al., 2022b), further refinement of patient selection criteria, optimal timing, and treatment combinations will be critical to maximizing the clinical benefit of SBRT in this setting. Future research may also explore synergistic combinations of SBRT with immunotherapy and identify predictive biomarkers of radiosensitivity, such as DNA damage repair alterations or immune gene expression profiles, to optimize patient selection and outcomes.

## 5 Conclusion

This systematic review and meta-analysis demonstrate that, in predominantly observational cohorts, SBRT demonstrates consistently high local control and a favorable safety profile for patients with OMOC. Across diverse clinical settings and patient populations, SBRT consistently achieved high LC rates with minimal toxicity, including in platinum-resistant and oligoprogressive contexts. These findings support the integration of SBRT into multidisciplinary management strategies for selected OMOC patients, particularly those with limited disease burden or under maintenance therapies. However, because our pooled estimates summarize heterogeneous, non-comparable populations, they should be viewed as descriptive and hypothesis-generating rather than definitive measures of comparative benefit. Data were insufficient for robust subgroup meta-analyses; prospective trials focused on well-defined scenarios are needed to validate these signals, to define optimal patient selection criteria, clarify the timing of intervention, and evaluate long-term oncologic benefits.

## Data Availability

The original contributions presented in the study are included in the article/supplementary material, further inquiries can be directed to the corresponding author.
